# A Case of Anorexia Nervosa with Focal Cortical Dysplasia

**DOI:** 10.1155/2024/7478666

**Published:** 2024-04-12

**Authors:** Hiroki Nemoto, Kazuo Imagawa, Takashi Enokizono, Yosuke Masuda, Masayuki Ide, Takuma Deguchi, Monami Hara, Atsushi Morita, Takahiro Kido, Mai Tanaka, Tatsuyuki Ohto, Hidetoshi Takada

**Affiliations:** ^1^Department of Pediatrics, University of Tsukuba Hospital, 2-1-1 Amakubo, Tsukuba, Ibaraki, Japan; ^2^Department of Child Health, Institute of Medicine, University of Tsukuba, 2-1-1 Amakubo, Tsukuba, Ibaraki, Japan; ^3^Department of Neurosurgery, Institute of Medicine, University of Tsukuba, 2-1-1 Amakubo, Tsukuba, Ibaraki, Japan; ^4^Center for Medical Sciences, Ibaraki Prefectural University of Health Sciences, 4669-2 Ami, Inashiki-gun, Ami-machi, Ibaraki, Japan

## Abstract

Anorexia nervosa (AN) is a fatal condition associated with extreme underweight and undernutrition. It is more common in young females, with a female-to-male ratio of 10 : 1. Focal cortical dysplasia (FCD) is characterized by dysplasia of the cerebral cortex and is a common cause of pharmacoresistant epilepsy. However, FCD associated with AN has never been reported. We report the first case of AN in a 12-year-old male diagnosed with FCD-type 2 on head magnetic resonance imaging (MRI). He became concerned about lower abdominal distention and began reducing his food intake. He was admitted to our hospital after weight loss of 10 kg in a 1 year. Head MRI showed a localized high-signal area from the cortex to the white matter of the fusiform gyrus near the left hippocampus, with no associated decreased blood flow or electroencephalography (EEG) abnormalities. These findings were characteristic of FCD type II. In males with AN, the search for underlying disease is particularly important. The pathophysiology of the association between AN and FCD is unclear. However, both conditions are reportedly associated with autism spectrum disorder. Further cases are needed to clarify whether FCD is associated with eating disorders.

## 1. Introduction

Anorexia nervosa (AN) is an eating disorder characterized by food restriction and body image disturbance [1]. It may cause multiorgan dysfunction and has a high mortality rate [2]. AN is more common in young females than in males [3]. Although various risk factors, including genetic and metabolic factors, have been postulated, none have been clearly determined [4]. Head imaging studies should be conducted to rule out the possibility of an intracranial lesion, such as a brain tumor, as the cause of AN. The preoccupation with eating behaviors and body shape seen in AN is similar to that seen in autism spectrum disorder (ASD). The combination of AN and ASD is not uncommon [5].

Focal cortical dysplasia (FCD) is characterized by dysplasia in the cerebrum and causes pharmacoresistant epilepsy [6, 7]. FCD results from focal developmental abnormalities in the cerebral cortex, which are associated with several molecular abnormalities related to cell adhesion [8]. FCD has reportedly been implicated in neurodevelopmental disorders and ASD [9]. However, FCD associated with AN has never been reported. We report the first case of AN in a 12-year-old male with FCD.

## 2. Case Presentation

A 12-year-old male with no specific birth or medical history became concerned about lower abdominal distention and began reducing his food intake 3 months prior to consultation. He exercised obsessively according to his food intake. Although he was considerably losing weight, he commented that his belly was fat and did not want it to get any fatter. Body image disturbances and anxiety regarding being overweight were observed. During his initial consultation, he was very lean, with a body mass index (BMI) of 11.5 kg/m^2^. He also had sinus bradycardia with a heart rate of approximately 40 beats/min. We attempted to improve his weight with enteral nutritional supplements while identifying underlying diseases by head magnetic resonance imaging (MRI) and abdominal ultrasound. However, he became disoriented and was emergently hospitalized 7 days after his first visit. On admission, he was 145 cm tall, weighed 20.9 kg (BMI: 9.9 kg/m^2^), and presented with impaired consciousness with Glasgow Coma Scale (GCS) E3V4M6, bradycardia with a heart rate of about 40 beats/min, and slow breathing with a respiratory rate of about 10 cycles/min. Blood gas analysis and blood test revealed hypercapnia, hypoglycemia, and elevated levels of creatinine, blood urea nitrogen, and transaminases (Table 1). His movements were slow but not paralytic and his cranial nerves were normal. As his hypoglycemia and dehydration were chronic, rapid correction was avoided to prevent the occurrence of refeeding syndrome, heart failure, pleural effusion, and ascites. Thus, we slowly corrected the hypoglycemia and dehydration (glucose infusion rate: 0.6–1.0 mg/kg/min; water: 20 mL/kg/day). On the second day of admission, he developed impaired consciousness, with GCS E1V1M1 and low blood pressure; he was transferred to the intensive care unit (ICU). The impaired consciousness and circulatory collapse were attributed to the marked hypoglycemia and dehydration. After these were corrected, his consciousness improved, and he was transferred to the general ward from ICU on the third day of hospitalization. On the fourth day of admission, his hepatic transaminases reached the peak levels, as follows: aspartate transaminase (AST), 4,782 U/L; and alanine transaminase (ALT), 2,265 U/L. Coagulopathy (prothrombin time (%): 28%) was also observed. An acute liver failure-like condition was considered. He was provided liver support therapy, including glucose infusion and enteral nutrition, and the AST and ALT levels were gradually improved.

**Figure 1:**
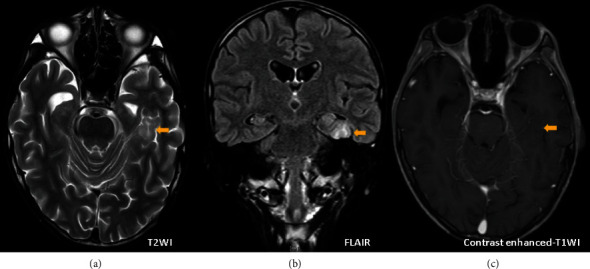
Head MRI was performed 6 weeks after admission. (a) T2-weighted image, (b) FLAIR, and (c) contrast enhanced-T1 weighted image (right) are shown. T2-weighted and FLAIR images show a localized high-signal area from the cortex to the white matter of the fusiform gyrus near the left hippocampus (arrows). It is accompanied by gray matter swelling and subcortical white matter atrophy. There was no contrast enhancement. MRI, magnetic resonance imaging; FLAIR, fluid attenuated inversion recovery.

A head MRI scan was performed 6 weeks after admission when his general condition was stable. T2-weighted and fluid-attenuated inversion recovery images showed a localized high-signal area from the cortex to the white matter of the fusiform gyrus to the parahippocampal gyrus near the left hippocampus (Figure 1). The cortex in high-signal lesions is thickened, the boundary between white matter and cortex is indistinct and localized, and there is no tendency for invasion into the surrounding white matter. There was no contrast enhancement, and the imaging findings were characteristic of FCD type II. Electroencephalography and cerebral blood flow scintigraphy showed no significant results.

Once the patient's physical condition was stabilized, we administered several psychological tests. Wechsler Intelligence Scale for Children, Fourth Edition, showed his full-scale intelligence quotient (IQ) to be 119, which was above average for his age group (scores for each index are as follows: verbal comprehension index, 117; perceptual reasoning index, 115; working memory index, 123; and processing speed index, 99). The synthetic House-Tree-Person test showed his characteristics as low-internal energy and lack of personality richness and positivity. The Rorschach test indicated that he had a high level of vigilance and marked anxiety as prominent psychological traits. It suggested susceptibility to impairment in psychological functioning when confronted with complex or ambiguous situations. His thoughts and values lacked flexibility, showing a preference for routine work and a tendency to resist change. In addition, his mother noted that he was very particular, for example, he followed the school schedule even on days when school was not in session, which could be considered ASD tendencies. However, although his communication with others was unique, it was not to the extent that it interfered with his daily life and did not meet the criterion A of ASD according to Diagnostic and Statistical Manual of Mental Disorders, Fifth Edition (DSM-5) [10].

**Figure 2:**
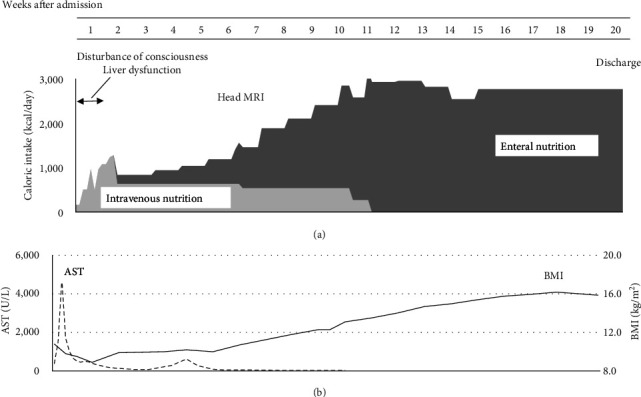
Posthospitalization progress. (a) Calorie intake and (b) AST and BMI. In the first week after admission, disturbance of consciousness and liver dysfunction appeared. The patient was treated first with intravenous and then with enteral nutrition, and his organ damage gradually resolved. AST, aspartate transaminase; BMI, mody mass index.

Owing to the patient's poor general condition on admission, his caloric intake was increased gradually using intravenous nutrition before starting enteral nutrition. The intake could be titrated without causing electrolyte abnormalities that are known to occur as refeeding syndrome (such as hypophosphatemia or hypokalemia). After the resolution of intestinal edema, which was confirmed by sonography, enteral nutrition was initiated and increased gradually. Although he never threw up or had self-induced vomiting, he had a very slow oral intake and was unable to reach the target oral intake, so he was fed through a nasogastric tube. After being counseled that his life was in danger due to his extreme emaciation, his oral intake gradually increased and the nasogastric tube was discontinued by week 9. BMI decreased to 8.9 kg/m^2^ after admission, but weight gain was steady after enteral feeding resumed. By the 12^th^ week of hospitalization, his BMI recovered to 13 kg/m^2^. By the 20^th^ week, it was 15; therefore, he was discharged (Figure 2). His BMI remained stable at approximately 16 kg/m^2^. Once the patient's condition was physically stabilized, psychiatric counseling and disease education were initiated. The patient has been followed up for about 2 years to date without any recurring eating disorders or weight loss. On follow-up, neither morphological changes on imaging nor the onset of epilepsy were noted.

## 3. Discussion

To date, AN with FCD has never been reported, and this case suggests a link between these conditions.

AN is a potentially fatal condition that causes systemic organ damage due to the patient being extremely underweight and undernourished because of poor feeding. Young females are more frequently affected; male patients show only one-tenth of the frequency seen in female patients [11]. If a male patient is suspected to have AN, the possibility of having underlying diseases might be higher. AN is diagnosed by meeting the following three criteria [10]: (a) restriction of energy intake relative to requirements, leading to considerably low body weight in the context of age, sex, developmental trajectory, and physical health; (b) intense fear of gaining weight or becoming fat or persistent behavior that interferes with weight gain, even at a remarkably low weight; and (c) disturbance in how one's body weight or shape is experienced, undue influence of body weight or shape on self-evaluation, or persistent lack of recognition of the seriousness of the current low body weight. This patient presented with remarkable weight loss due to poor feeding, which was triggered by an excessive concern over a protruding lower abdomen. He also displayed overactivity and refusal to eat to avoid gaining weight. He had body image disturbance and obesity fears but not a schizophrenic or depressed mental state. Physical conditions causing weight loss, such as diabetes, hyperthyroidism, malignancy, and inflammatory bowel disease, were negative. Thus, he was diagnosed with AN. Although gender dysphoria may be accompanied with AN in males, our patient's gender identity conformed with the sex he was assigned at birth.

FCD is characterized by dysplasia of the cerebral cortex. The cerebral cortex has a characteristic six-layer structure, formed during development by neurons migrating in-side-out from the periventricular regions [12]. This migration of neurons requires the molecules, such as N-cadherin and reelin, which are involved in adhesion, and the abnormalities in these molecules can cause FCD [8]. FCD is the most frequent cause of pharmacoresistant epilepsy requiring surgical treatment and is associated with hippocampal sclerosis and neoplastic disease [13]. FCD is classified according to the direction of cortical neuronal array abnormalities, presence or absence of dysmorphic cells, and development of hippocampal sclerosis and neoplastic lesions. In almost all type IIb and approximately 30% of type IIa cases, characteristic findings on head MRI confirm the diagnosis [14]. In this case, the lesion was discovered incidentally on head MRI while investigating an underlying disease. Its MRI features indicated it was FCD type IIb. Possible differential diagnoses based on head MRI findings include neuronal migration disorders other than FCD and various brain tumors, especially gray matter heterotopia or low-grade gliomas. In the present case, FCD is mostly suspected because of the localized nature of the lesion and the absence of abnormal findings in the subcortical white matter. Follow-up MRI was performed, but there was no trend toward progression for 2 years, which supports the diagnosis of FCD, but not brain tumor.

Comorbidity of FCD and AN has not been reported to date, but the common factor of ASD may provide a consistent potential explanation. The fact is that the frequency of AN and ASD complications is relatively high. The previous report showed that approximately 18% of AN cases are complicated by ASD [4]. Cognitive features observed in AN include a strong preoccupation with weight and body shape, inadequate awareness of the serious dangers of being underweight, and values directly influenced by body shape, making it difficult to hold other values. Previous reports have linked somatoform disorders to the inferior portion of the medial temporal lobe, in which FCD was also seen in this case. It is possible that AN may have developed in a localization-dependent manner [15]. These cognitive characteristics are similar to those of ASD, such as impaired social skills and patterned behavior. On the other hand, there is no literature showing a link between FCD and AN. However, mutations in reelin, a protein that is a cause of FCD and regulates neuronal migration and positioning, have been reported to be associated with the development of ASD. It has been suggested that abnormalities in reelin, which is required for normal neural migration, cause ASD [9]. Therefore, it might be possible that FCD and AN are indirectly related in the pathophysiology. The patient's overall IQ was above average. Although it was not appropriate to confer a concrete ASD diagnosis upon him, his low conceptualization skills and poor implicit comprehension indicated ASD-related tendencies. The development of his body image disturbance was not triggered by his esthetic evaluation or comparison with others. He was concerned about the issue of obesity being bad for health, as he had learned at school. This indicates that he took words literally, a characteristic compatible with ASD. Therefore, the early intervention with appropriate disease education alone enabled him to resume proper eating habits.

In this case, FCD was not localized in the region where the feeding center is generally located, and its direct relationship to the eating disorder is still being determined. However, with regard to the fusiform gyrus, where FCD was present in this case, studies using functional MRI have suggested that this region is dysfunctional in patients with ASD [16]. The fusiform gyrus has been reported to be associated with ASD because it plays an essential role in face perception [17]. The fusiform gyrus plays an important role in face and body perception so that body image impairment may have occurred. The previous reports showed that functional MRI in patients with AN identified fusiform gyrus dysfunction [18]. These suggest that FCD may be involved in AN and ASD tendencies. The fusiform gyrus is also a part of a system that plays a central role in face and body perception, and abnormalities in this region may have contributed to the body image disturbance [19]. The localization of FCD in this case may have contributed to both ASD and AN. This case indicates the possibility that FCD is associated with AN with some pathophysiological background. It was a single case report, and the findings cannot be generalized. Further case series are needed to clarify whether FCD is associated with eating disorders.

In our patient, severe liver damage occurred. In AN, 12%–41% are associated with liver damage, and younger age (<30 years), low BMI (<12 kg/m^2^), and hypoglycemia are known risk factors [20]. The possible mechanism is that the increased autophagy in the liver and ischemic hepatitis due to decreased blood flow, and hepatic dysfunction may also occur in patients with refeeding syndrome [21]. Because this patient was considered at high risk for refeeding syndrome, we paid close attention to electrolyte abnormalities including phosphorus and the appearance of cardiac failure and pleural effusion due to fluid overload, and managed the patient by avoiding hyperglycemia and reducing fluid administration. There were no electrolyte abnormalities such as hypophosphatemia at the time of impaired consciousness and acute hepatic dysfunction; thus, refeeding syndrome was unlikely the cause. However, this patient showed marked hypoglycemia at the time of impaired consciousness, which improved with glucose administration, followed by frequent electrolyte monitoring with attention to signs of heart failure and increased sugar and fluid administration, which improved liver dysfunction. This suggested that hypoglycemia and hypoperfusion were the cause of impaired consciousness and liver dysfunction.

## 4. Conclusion

This is the first case report that suggests a link between AN and FCD. Further cases are needed to clarify whether FCD is directly associated with eating disorders. And it may help to improve understanding of the pathophysiology of eating disorders in children with ASD tendencies.

## Figures and Tables

**Table 1:** Blood test findings on admission.

Complete blood count		Biochemistry			Coagulation		
WBC	9,000 /*μ*L	AST	377	U/L	PT-INR	1.8	—
Seg	52.0 %	ALT	298	U/L	APTT	28.9	s
Band	0.0 %	LDH	513	U/L	Fibrinogen	121	mg/dL
Lym	39.0 %	T-Bil	2.1	mg/dL	—	—	—
Mono	9.0 %	Na	150	mEq/L	—	—	—
Hb	13.9 g/dL	Cl	111	mEq/L	—	—	—
Ht	42.0 %	K	3.8	mEq/L	—	—	—
MCV	83.8 fL	BUN	73.7	mg/dL	—	—	—
MCH	27.7 pg	Cre	0.84	mg/dL	—	—	—
MCHC	33.1 %	CK	347	U/L	—	—	—
Plt	86 × 10^3^ /*μ*L	Ca	9.0	mg/dL	—	—	—
—	—	IP	4.5	mg/dL	—	—	—

MCV, mean corpuscular volume; MCH, mean cell hemoglobin; MCHC, mean corpuscular hemoglobin concentration; AST, aspartate aminotransferase; ALT, alanine transaminase; LDH, lactate dehydrogenase; BUN, blood urea nitrogen; CK, creatine kinase; PT-INR, prothrombin time-international normalized ratio; APTT, activated partial thromboplastin time.

## Data Availability

The patient's details are anonymized and thus unavailable.
